# UFMylation System: An Emerging Player in Tumorigenesis

**DOI:** 10.3390/cancers14143501

**Published:** 2022-07-19

**Authors:** Yu Jing, Ziming Mao, Fengling Chen

**Affiliations:** Department of Endocrinology and Metabolism, Shanghai Ninth People’s Hospital, Shanghai JiaoTong University School of Medicine, Shanghai 200025, China; jingyu_1031@sjtu.edu.cn (Y.J.); maoziming@sjtu.edu.cn (Z.M.)

**Keywords:** UFMylation, UFM1, tumorigenesis, post-translational modifications, ubiquitin-like molecules

## Abstract

**Simple Summary:**

The ubiquitin-fold modifier 1 (UFM1) is a newly identified post-translational modification protein that has been implicated in multiple cellular processes and diseases. Noticeably, an aberrant UFM1 modification system has been closely related to various types of tumorigeneses, implying that the restoration of UFMylation homeostasis may serve as a promising therapeutic strategy. In this review, we summarize the structure, process and biological functions of the UFM1 modification system. In particular, we discuss the relationship between the UFMylation system and tumorigenesis, illustrating the underlying mechanisms and future perspectives. This article aims to improve our understanding of UFM1 modification, as well as provide some new strategies for cancer treatment.

**Abstract:**

Ubiquitin-fold modifier 1 (UFM1), a newly identified ubiquitin-like molecule (UBLs), is evolutionarily expressed in multiple species except yeast. Similarly to ubiquitin, UFM1 is covalently attached to its substrates through a well-orchestrated three-step enzymatic reaction involving E1, the UFM1-activating enzyme (ubiquitin-like modifier-activating enzyme 5, UBA5); E2, the UFM1-conjugating enzyme 1 (UFC1); and E3, the UFM1-specific ligase 1 (UFL1). To date, numerous studies have shown that UFM1 modification is implicated in various cellular processes, including endoplasmic reticulum (ER) stress, DNA damage response and erythroid development. An abnormal UFM1 cascade is closely related to a variety of diseases, especially tumors. Herein, we summarize the process and functions of UFM1 modification, illustrating the relationship and mechanisms between aberrant UFMylation and diversified tumors, aiming to provide novel diagnostic biomarkers or therapeutic targets for cancer treatments.

## 1. Introduction

Post-translational modifications (PTMs) refer to covalent or enzymatic modifications of proteins during or after protein biosynthesis. Ubiquitin and ubiquitin-like molecules (UBLs) have been identified as the third most common type of post-translational modification after phosphorylation and glycosylation [1], and they play pivotal roles in fine-tuned cellular activities and communications. Ubiquitin-fold modifier 1 (UFM1), one of the newly identified UBLs, was initially discovered in 2004. Although extensive studies have been performed on the ubiquitin system, the biological functions and working mechanisms of UFM1 modification remain elusive.

**Figure 1:**
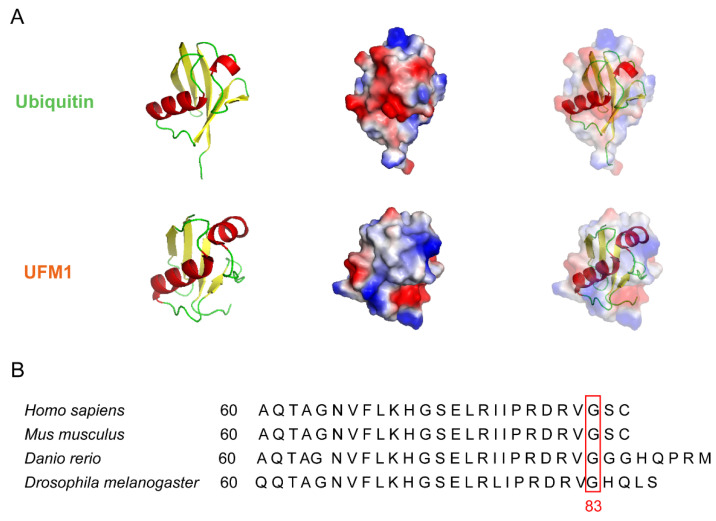
Structure and sequence alignment of UFM1. (**A**) Comparison between 3D structures and the electrostatic potential surfaces of ubiquitin-fold modifier 1 (UFM1) and ubiquitin (Ub). PDB IDs for ubiquitin and UFM1 are 1UBQ and 5IA7, respectively. Left panel—3D structure, α-helices and β-strands are shown in red and yellow, respectively. Middle panel-electrostatic potential surface, positive and negative potentials are shown in blue and red, respectively. Right panel—the merged image. (**B**) Sequence alignment of UFM1 in different species. The 83 amino acid residue in all species is indicated by red box.

UFM1, containing 85 amino acids (precursor form), is evolutionary expressed in multiple species, except yeast. Although UFM1 has only 16% sequence identity similarity to ubiquitin, it displays a conserved tertiary structure of a ubiquitin fold with specific β-sheets and an α-helix (Figure 1A) [2,3]. As with ubiquitin and other UBLs, matured UFM1 (83 amino acids) ends with a C-terminal Glycine (Gly), which forms an isopeptide bond with the target proteins. However, in contrast to ubiquitin and other UBLs that contain two Gly residues at the C-terminus, UFM1 has a Val residue instead of the first Gly (Figure 1B) [4]. Recent studies have revealed that UFM1 modification is closely related to a range of cellular processes, such as endoplasmic reticulum (ER) stress, hematopoiesis, fatty acid metabolism, transcriptional regulation, neurodevelopment, and the DNA damage response [5]. In addition, an abnormal UFM1 cascade is implicated in a variety of diseases, including diabetes [6,7], heart failure [8,9], inflammatory disease [10], liver development [11], hip dysplasia [12], cancer [13] and brain development [14,15,16]. However, the physiological and pathological roles of UFM1 modification in different types of cancers are not completely understood. In addition, whether the UFM1 cascade could be a potential therapeutic target for cancer diagnosis and treatment remains unknown. In this review, we summarize the previous studies on the UFMylation and tumorigenesis, aiming to give a comprehensive overview of the current knowledge on UFM1 modification and tumor development, as well as highlighting the knowledge gaps and the future perspectives on the UFM1 modification in the cancer field.

## 2. The UFM1 (De-)Conjugation System

**Figure 2:**
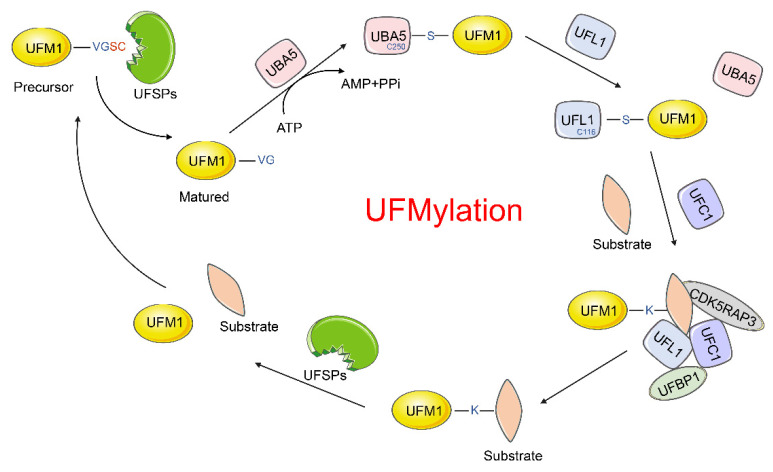
The UFM1 conjugation system. The precursor form of UFM1 is cleaved by UFSPs to expose its C-terminal conserved Gly residue. Then, matured UFM1 is activated by UBA5 that consumes ATP, forming a high energy thioester bond with Cys250 of UBA5. UFC1 next binds to UBA5 and retrieves UFM1 from UBA5 by forming a thioester bond with UFM1. Finally, UFC1, together with UFL1, transfer UFM1 to its substrate. Both UFBP1 and CDK5RAP3 are possible adaptor proteins that allow the ligase UFL1 to recruit a wider pool of substrates. Additionally, since UFMylation is a reversible process, the UFM1 molecules can be removed from their targets by UFSPs. Abbreviation: VGSC is an amino acid motif (valine–glycine–serine–cysteine). S indicates the thioester bond. K represents the lysine residue of the substrate.

Firstly, the UFM1 precursor form must be cleaved by the UFM1-specific cysteine proteases (UFSP1 and UFSP2) [17,18,19], which cleave the C-terminal dipeptide Ser–Cys to expose the single conserved Gly residue [2,17]. Then, similarly to ubiquitination, the matured UFM1 is conjugated to target proteins by an orchestrated three-step enzymatic reaction involving E1, the UFM1-activating enzyme (ubiquitin-like modifier-activating enzyme 5, UBA5), E2, the UFM1-conjugating enzyme 1 (UFC1), and E3, the UFM1-specific ligase 1 (UFL1). Although the ubiquitination system consists of tens of different E2s and hundreds of varieties of E3s, only one of each enzyme has been identified in the UFMylation system [4]. In addition, this modification process also requires the activation of ATP. Consequently, with the help of ATP, the matured UFM1 is activated by UBA5. With a series of adenylation and thioesterification reactions, UFM1 forms a high energy thioester bond with the Cystine (Cys) 250 of UBA5. Then, UFC1 interacts with the UFC1-binding sequence (UBS, amino acid sequence 392–404) of UBA5, and the activated UFM1 is transferred to UFC1 via a trans-esterification reaction, forming a thioester linkage with Cys116 in UFC1. Finally, UFC1, together with UFL1, transfers UFM1 to its target proteins [13]. Meanwhile, the specific UFSPs (UFSP1 and UFSP2) can also reverse this process, removing UFM1 from its target proteins (Figure 2) [17].

### 2.1. UBA5

UBA5, also known as UBE1DC1, belongs to the non-canonical E1 enzyme family, which includes ATG7 and UBA4. Unlike the typical E1 enzymes composed of the first and second catalytic cysteine half-domains (FCCH and SCCH), the adenylation domain and the C-terminal ubiquitin-fold domain [20], UBA5 lacks the FCCH and SCCH domains, but instead contains an adenylation domain harboring the catalytic cysteine [21]. Therefore, UBA5 is significantly smaller than the other E1 enzymes. Generally, UBA5 has two isoforms, with the difference being the additional 56 amino acids in the N-terminal extension adjacent to the adenylation domain, which is supposed to be important for ATP binding [22]. Traditionally, UBA5 is mainly expressed in the cytoplasm, and forms a homodimer through its adenylation domain. In contrast to the canonical E1, which activates UBLs through a three-step mechanism, UBA5 activates the UFM1 via a two-step mechanism [23]. Firstly, at the expense of ATP, the adenylation domain of UBA5 attacks and activates the C-terminal glycine residue of UFM1, generating the adenylated UFM1. Then, UFM1 is conjugated to Cys250 of UBA5 via a thioester bond formation [2]. Once UFC1 interacts with the UBS domain of UBA5, it leads to the transfer of UFM1 from UBA5 to the Cys116 of UFC1 by a trans-esterification reaction. Moreover, it is also reported that UBA5 can activate SUMO2, another ubiquitin-like protein [24], but the specific mechanism remains to be identified.

A previous study revealed that *Uba5*-deficient mice died in utero owing to severe anemia combined with the defective differentiation of both megakaryocytes and erythrocytes [25]. Meanwhile, through whole-exome sequencing, several studies have identified that biallelic variants in UBA5 resulted in early onset or severe infantile-onset encephalopathy due to the disruption of the UFM1 modification pathway [26,27]. UBA5 mutations are also associated with autosomal recessive cerebellar ataxia and early onset epileptic encephalopathy [14,28,29]. Additionally, a recent study found that a homozygous UBA5 pathogenic variant could cause a fatal congenital neuropathy [30], suggesting the crucial role of UBA5 in neurodevelopment.

### 2.2. UFC1

UFC1, also known as HSPC155, consists of 167 amino acids (molecular weight: 19.4 kDa), and is mainly localized in the nucleus, and only partially in the cytoplasm [2]. UFC1 shares poor sequence homology with other E2 enzymes, with the exception of the catalytic core domain where approximately 10 amino acid residues encompass the active site Cys116 residue, forming a flexible loop that is highly solvent-accessible [31]. Similar to the ubiquitin E2 enzymes, the active site Cys116 residue catalyzes the trans-esterification of UFM1 during its transfer from UBA5. Through structural studies, researchers discovered that the N-terminal helix of UFC1, which is not present in other E2 enzymes, can adopt a variety of conformations, most likely to accommodate different substrates, thus conferring thermal stability to the substrates [31,32,33]. Moreover, NCAM140, a neural cell adhesion molecule, is found to interact with UFC1, and in the presence of UFM1, the endocytosis of NCAM140 also increased, suggesting a potential novel function of UFMylation for cell surface proteins [34]. Although the physiological and pathological role of UFC1 is poorly understood, its long non-coding RNA (LncRNA) and long intergenic RNA (lincRNA) are reported to regulate the progression of numerous cancer types [35,36,37,38].

### 2.3. UFL1

Similarly, to date, UFL1, also known as Maxer, NLBP, KIAA0776 and RCAD, is the only known E3 enzyme that transfers UFM1 to the lysine residue of target proteins. UFL1 has a transmembrane domain and a nuclear localization signal (NLS), and mainly localizes in the endoplasmic reticulum (ER) membrane [21,39]. Initially identified as a UFBP1-interacting protein [40], UFL1 does not possess any typical domains conserved in E3 ligases, such as the homologous to the E6AP carboxyl terminus domain (HECT), the really interesting new gene (RING) finger domain or the U-box domain [2]. Instead, it contains a highly conserved N-terminal domain that is vital for UFM1 transfer to substrate proteins [40]. Thus, UFL1 seems to function as a scaffold protein that recruits the E2 enzyme and target proteins akin to RING type Ubiquitin E3 ligase. To date, UFL1 has been reported to catalyze a variety of UFMylation substrates, and the most well-known substrate is UFBP1 [7]. Notably, in the process of the UFMylation of activating signal cointegrator 1 (ASC1), a nuclear receptor co-activator, the E3 ligase activity of UFL1 also requires the assistance of UFBP1, suggesting that the UFMylation of substrates may require not only UFL1, but also the other proteins [41]. Meanwhile, several studies have revealed that UFL1 forms a complex with CDK5RAP3 (also known as C53 and LZAP) and UFBP1 proteins, which may be potentially involved in the pathogenesis of spinocerebellar ataxia [39,42,43]. Thus, UFBP1, together with CDK5RAP3, is regarded as a satellite component and key regulator of the UFMylation system.

Notably, several studies have begun to uncover the biological functions of UFL1. Zhang et al. [44] discovered that both the germ-line and somatic deletion of *Ufl1* led to impaired hematopoietic development and embryonic lethality. Meanwhile, Cai et al. [10] reported that the ablation of either UFL1 and UFBP1 resulted in a significant loss of both Paneth and goblet cells, which in turn led to dysbiotic microbiota and increased susceptibility to colitis. Meanwhile, cardiac specific *Ufl1*-knockout mice developed cardiomyopathy and heart failure [8]. Additionally, UFL1 was implicated in pancreas amylase secretion and ER homeostasis [45]. Additionally, *Ufl1* deficiency-induced kidney atrophy was associated with the disruption of ER homeostasis [46]. Nonetheless, the biological functions of UFL1 in disease pathogenesis remain to be fully elucidated.

### 2.4. UFSP1 and UFSP2

The conjugates are cleaved by the action of specific UFSPs (UFSP1 and UFSP2). Both UFSP1 and UFSP2 belong to a novel cysteine protease subfamily, simply because they show no sequence homology compared with the most deubiquitinating enzymes (DUBs) and ubiquitin-like protein-specific proteases (ULPs), but possess a catalytic triad universally shared by cysteine proteases [18,19]. The UFSPs mediate the UFM1 maturation step as well as the de-UFMylation process. UFSP1 has a papain-like fold with a unique active site consisting of a conserved Cys box and a conserved Asp-Pro-His box, instead of the classical Cys-His-Asp catalytic triad [18]. UFSP2 differs from UFSP1 due to the presence of an extended N-terminal domain, which may play a critical role in recognizing specific substrates during the deconjugation process [19]. It should be noted that human UFSP1 seems to be catalytically inactive due to the lack of N-terminus and therefore, UFSP2 is the main deUFMylase in humans [17] which resides in both the nucleus and the cytoplasm.

## 3. The Physiological Function of UFMylation

### 3.1. ER Stress

The endoplasmic reticulum (ER) is an organelle that plays a major role in the synthesis, folding and maturation of approximately one third of the proteome and most of the secreted and transmembrane proteins in the plasma membrane [47]. It also regulates the metabolic processes, including gluconeogenesis and lipid synthesis, as well as maintaining intracellular calcium homeostasis [48]. Normally, the ER functions with a set of sophisticated folding and modifying machinery led by numerous ER enzymes, however, due to various pathological conditions, the abnormal modification and misfolding of proteins still regularly occurs and they accumulate in the ER, which ultimately activates the ER stress [48,49]. As a response to ER stress, cells constantly initiate protein quality-control systems, including the unfolded protein response (UPR), ER-associated degradation (ERAD) and autophagy pathways [50]. URR activates a signaling pathway involving three sensors, namely inositol-requiring enzyme 1α (IRE1α), protein kinase-like ER kinase (PERK), and activating transcription factor 6 (ATF6) [51]. If UPR fails to overcome ER stress, apoptotic responses will be activated. Then, through the ERAD process, unfolded proteins are removed to the cytosol for the subsequent ubiquitylation and degradation by the 26S proteasome [52].

Interestingly, Azfer et al. [9] first reported that UFM1, as well as ER stress-related genes, were transcriptionally upregulated during the development of ischemic heart disease. Lemaire et al. [7] further discovered that the expressions of UFM1, UFBP1 and UFL1 were significantly elevated in the ER stress-induced beta-cell line INS-1, while the knockdown of UFM1 or UFBP1 enhanced cell apoptosis upon ER stress. Likewise, the UFM1 modification system was transcriptionally increased by the inhibition of vesicle trafficking using brefeldin A (BFA), and the knockdown of the UFM1 modification system in U2OS cells triggered UPR and the amplification of the ER network [53]. Surprisingly, the luciferase reporter and ChIP assay illustrated that UFM1 might be modulated by XBP1, a crucial transcription factor in UPR, further implying a direct link between UFMylation and ER stress [53]. Similar phenomena were also found in diabetic mouse macrophages [54], the exocrine pancreas [45], kidney atrophy disease [46] and gut inflammation [10], indicating that the relationship between the UFM1 system and ER stress is a general and universal phenomenon. Mechanistically, UFL1 exerted a protective role on the pathogenesis of cardiomyopathy via regulating PERK signaling and consequently cardiomyocyte cell death [8]. In addition, the deficiency of *Ufl1* resulted in kidney atrophy due to the disruption of endoplasmic reticulum homeostasis [46]. Meanwhile, UFBP1 knockout caused the elevation of ER stress and the activation of UPR combined with the cell death program in intestinal epithelial cells, while the administration of a small molecular chaperone partially reversed these effects [10]. Recently, a study further explained that the depletion of UFBP1 could repress IRE1α-XBP1 signaling and activate the PERK-eIF2α-CHOP apoptotic pathway through the UFMylation modification of the ER-stress sensor IRE1α [55]. Consequently, UFM1 modification may exert a protective role in maintaining ER homeostasis and avoiding ER stress-induced apoptosis.

### 3.2. DNA Damage Response

Endogenous and exogenous DNA damaging factors such as ultraviolet (UV) light radiation, carcinogens and reactive radicals pose a serious hazard to the cellular genome’s integrity. To combat these threats, cells have evolved mechanisms termed the DNA damage response (DDR) to sense DNA lesions, signal their presence and promote their repair. Double-strand breaks (DSBs) are the most cytotoxic type of DNA lesion, which can repaired be either by homologous recombination (HR), a high-fidelity process, or by the error-prone process of non-homologous end-joining (NHEJ) [56]. Previous studies have shown that ataxia–telangiectasia mutated (ATM) is a predominant transducer of the DSBs response [57], which can be rapidly activated by the MRE11–RAD50–NBS1 (MRN) complex or acetyltransferase Tip60 after DSBs induction [58]. Intriguingly, MRE11, a member of the MRN complex, was discovered to be UFMylated on K282, and this UFMylation is required for the MRN complex formation, DSB-induced optimal ATM activation, HR repair, and the overall maintenance of genome integrity. Consistently, the mutation of MRE11 (G285C) identified in uterine endometrioid carcinoma exhibited a similar cellular phenotype as the UFMylation-defective mutant MRE11 (K282R) [59]. Notably, MRE11 UFMylation was also implicated in telomere shorting in *Ufm1* or *Ufl1* deficient zebrafish and UFL1 knockout Hela cells by interacting with NBS1 and subsequently the telomere protein TRF2 [60]. However, in contrast to the aforementioned study, no defects were found in DSBs repair regarding MRN complex formation, ATM activation and HR in UFL1 knockout cells, which may be partially due to differences in the cell lines. Furthermore, it was reported that histone H4 could be monoufmylated at K31 following DNA damage, which is important for methyltransferase Suv39h1 and acetyltransferase Tip60 recruitment and thus ATM activation. ATM, in turn, could also phosphorylate UFL1 at S462, which enhanced UFL1 E3 ligase activity and promoted ATM activation in a positive feedback loop [61]. In addition, the tumor suppressor p53 plays a pivotal role in the DSBs by halting the cell cycle and facilitating the DNA repair processes. The phosphorylation of ATM serine 15 could activate p53 and the subsequent DNA repair processes. Surprisingly, Liu et al. [62] revealed that p53 could also be covalently modified by the UFM1 modification system, which in turn stabilizes p53 by antagonizing its ubiquitination and proteasome degradation. The knockout of UFL1 or UFBP1 promoted cell growth and tumor formation by decreasing p53 protein expression, highlighting the important role of UFM1 modification in maintaining p53 stability.

### 3.3. Erythroid Development

Normally, hematopoiesis is a complex process organized by transcriptional factors or cytokines that promote the self-renewal, differentiation and survival ability of hematopoietic progenitors. However, the dysregulation of these cycles often leads to pathological conditions including anemia and leukemia. To date, multiple pieces of evidence have demonstrated that the UFM1 conjugation system is involved in erythroid development. For instance, *Uba5* knockout mice exhibited severe anemia, followed by death in utero [25]. Further study illustrated that the loss of UBA5 is associated with the defective differentiation of both megakaryocytes and erythrocytes, while the transgenic expression of UBA5 in the erythroid lineage rescued the *Uba5*-deficient embryos from anemia and prolonged their survival [25]. Meanwhile, both UFL1 and UFBP1 are successively reported to be related to erythroid development. Germ-line and somatic deletion of *Ufl1* in mice impeded hematopoietic development, combined with severe anemia, cytopenia and ultimately embryonic death [44]. Similar results were found in *Ufbp1* germ-line and somatic knockout mice [63]. Furthermore, UFL1 and UFBP1 both influence hematopoietic stem cell (HSC) function and erythroid differentiation. In addition, these genes exert an impact on erythroid development via regulating ER stress and UPR [44,63]. Intriguingly, UFBP1 also influences HSC differentiation through regulating the activating signal co-integrator 1 (ASC1), a known UFMylation substrate, and subsequently the erythroid transcription factors [63]. Additionally, CDK5RAP3, a binding protein of cyclin-dependent kinase 5 (CDK5) activator, is proposed to be an adaptor for UFL1. Liu et al. [64] reported that CDK5RAP3 is essential for epiboly and gastrulation in zebrafish. Additionally, Yang et al. [11] found that *Cdk5rap3* knockout mice displayed prenatal lethality with severe liver hypoplasia.

## 4. Aberrant UFMylation Contributes to Various Tumors

Given the essential role of UFMylation in regulating various biological processes, it is not surprising that UFMylation plays a crucial role in the context of tumorigenesis. To date, numerous aberrant UFM1 modifications have been identified in multiple cancers, and the role of UFMylation in tumorigenesis has not been systematically explored. Herein, we review the current literature and elucidate the role and underlying mechanisms, shedding light on the impact of the UFM1 conjugation system on tumor development.

### 4.1. Breast Cancer

Breast cancer is one of the most prevalent forms of cancer in women [65]. It has been well documented that estrogen receptor-positive (ER+) tumors constitute a large proportion of breast cancer cases [66]. Activating signal cointegrator 1 (ASC1), a transcriptional coactivator of estrogen receptor-α (ERα) as well as of other nuclear receptors, is identified as a UFMylation substrate. The polyufmylated ASC1 could act as a scaffold to enhance the association of p300, SRC1, and itself to the promoters of ERα target genes, and therefore promote the proliferation of a large subset of breast tumor cells [41]. An in vivo study found that ASC1 overexpression or UFSP2 depletion exacerbated ERα-mediated tumor formation, whereas tamoxifen treatment abrogated this effect. Moreover, the expression of the ASC1 mutant, an UFMylation defective form, or knockout of the UBA5 inhibited tumor growth, suggesting that the UFMylation of ASC1 is important for the transactivation of ERα and thus breast cancer development [41]. Currently, targeting ferroptosis is considered to be a novel anti-cancer strategy, especially in breast cancer. Yang et al. [67] discovered that metformin could induce ferroptosis in breast cancer cell lines and therefore suppress tumor growth. However, this process is independent of canonical AMPK signaling. Mechanistically, the anti-cancer effect of metformin was achieved by inhibiting the UFMylation of SLC7A11, a cysteine transporter critical for ferroptosis, implying that targeting the UFM1/SLC7A11 pathway could be a promising cancer treatment strategy. Meanwhile, the E1 activating enzyme UBA5 was found to be upregulated in breast cancer and associated with poor prognosis. Its inhibitor, usenamine A, a natural product from Usnea longissimi, could induce apoptosis, autophagy and ER stress in breast cancer cells, decelerating the pathogenesis of breast cancer [68]. Additionally, the LncRNA of UFC1 promoted the proliferation and migration of breast cancer via the miR-34a/CXCL10 axis [37].

### 4.2. Gastric Cancer

The UFM1 conjugation system is also implicated in the pathogenesis of gastric cancer. In a study, the level of UFM1 was significantly decreased in gastric cancer tissue compared with the normal control [69]. Moreover, UFM1 was associated with the TNM stage and overall survival rate. The overexpression of UFM1 inhibited the oncogenic properties of gastric cancer both in vivo and in vitro, while the knockdown of UFM1 yielded the opposite results [69]. Further studies revealed that UFM1 increased the ubiquitination level of PDK1 and decreased the protein expression of PDK1, thus inhibiting PI3K/AKT signaling [69]. Similarly, Lin et al. [70] reported that the low expression of UFBP1 and CDK5RAP3 may indicate a worse outcome for gastric cancer patients. Moreover, UFBP1 is also related to drug sensitivity, i.e., the high expression of UFBP1 predicted a better prognosis in gastric cancer patients treated with platinum-based chemotherapy agents. UFBP1 could enhance the sensitivity of the gastric cancer cells to cisplatin by mediating the K48-linked polyubiquitin of the oxidative stress-response transcription factor Nrf2 and promoting its proteasome-mediated degradation. This effect further downregulated the expression of target gene aldo-keto reductase 1C (AKR1C), suggesting that UFBP1 may serve as a promising biomarker for chemotherapy in patients with gastric cancer [71]. Meanwhile, CDK5RAP3, the satellite component of the UFMylation system, has been shown to suppress the development of gastric cancer via inhibiting the phosphorylation of Akt/GSK-3β and negatively regulating Wnt/β-catenin signaling [72,73]. Moreover, the LncRNA of UFC1 promoted gastric cancer development by regulating the miR-498/Lin28b pathway [38].

### 4.3. Colon Cancer

Through the genomic profiling of the UFMylation family genes (UBA5, UFC1, UFL1, UFBP1, UFM1, UFSP1, UFSP2, and CDK5RAP3) across the Cancer Genome Atlas (TCGA) data cohort, researchers found that these genes have a high frequency of somatic copy number alterations (SCNAs). Among these genes, UFSP2 has the highest alteration score and is frequently deleted in 14 cancers [74]. Human tissue microarrays further confirmed that levels of UFSP2 were significantly decreased in colon cancer patients. The knockdown of UFSP2 promoted the growth of colon cancer cells and xenograft tumors [74]. GSEA analysis revealed that UFSP2 was mainly correlated with the DNA replication, cell cycle, spliceosome, ribosome, and mismatch repair processes, which is consistent with previous studies [61,62,75]. Moreover, biological experiments further validated that UFSP2 increased the expression of marker genes that were involved in the aforementioned processes, suggesting that UFSP2 may function as a tumor suppressor in colon cancer [74]. In addition, the long intergenic noncoding RNAs (lincRNAs) of UFC1, a subgroup of lncRNA, exerted the pro-proliferation and anti-apoptosis effects in colorectal cancer by regulating β-catenin and p38 signaling, which could be a potential therapeutic target and novel molecular biomarker for colorectal cancer [35].

### 4.4. Hepatocellular Carcinoma

Hepatocellular carcinoma (HCC) is one of the most common causes of cancer-related death worldwide. Risk factors for HCC include chronic hepatitis, alcohol addiction, metabolic liver disease, and exposure to dietary toxins [76]. Recently, emerging evidence demonstrated that the UFMylation conjugation system is related to HCC development. Chen et al. [77] discovered that the UFM1 expression was significantly lower in tumor tissues than that in adjacent tissues. Meanwhile, the lncRNA B3GALT5-AS1 functioned as an HCC suppressor by regulating the miR-934 and UFM1 pathway, therefore inhibiting HCC cell proliferation, invasion and metastasis. However, the direct role of UFM1 in HCC remains to be further investigated. Additionally, UFL1 was identified as a tumor suppressor in hepatocellular carcinoma through preventing cell invasion, inhibiting NF-kB signaling and increasing the stability of the LZAP protein, implying a critical role of UFL1 in the pathogenesis of HCC [43]. Nonetheless, the exact role of CDK5RAP3 in HCC remains controversial. A study reported that CDK5RAP3 may be an oncogenic gene, promoting cell migration and invasion properties in SMMC-7721 and HepG2 cell lines [78,79]. On the contrary, another group revealed that CDK5RAP3 functioned as a tumor-suppressor in HepG2 and sk-Hep1 cells [80]. Further works are still needed to explore the precise role, function, and mechanism of CDK5RAP3 in HCC.

### 4.5. Lung Cancer

Despite the tumor suppressor role of UFL1 in hepatocellular carcinoma, it may function as an oncogenic gene in lung cancer, specifically, lung adenocarcinoma. UFL1 was upregulated in cancerous tissue in the early stages of lung adenocarcinoma, while the overexpression of UFL1 promoted the proliferation of lung adenocarcinoma H1299 cells. UFL1 could bind to the regulatory domain of p120 catenin, and this binding inhibited the ubiquitin-mediated proteasome degradation of p120 catenin [81]. Differences in cancer types and substrates may partly explain the conflicting roles of UFL1 in hepatocellular carcinoma and lung cancer. Meanwhile, one of the UBA5 inhibitors derived from adenosine 5′sulfamate (ADS) was demonstrated to reduce the proliferation of lung cancer cells, highlighting the possible implications of the UBA5 inhibitor for cancer therapy [82]. Moreover, CDK5RAP3 was found to be elevated in lung adenocarcinoma tissue, and it may be a potential biomarker, but the specific role and mechanism need further study [83].

### 4.6. Others

In addition to the aforementioned tumors, the UFM1 conjugation family was also reported in other human carcinomas. For instance, chemoproteomic screening identified that UBA5 inhibited the development of pancreatic cancer in vivo and in vitro, which could be a novel strategy for treating pancreatic cancer [84]. Moreover, UFBP1 decelerated cell proliferation, migration and invasion properties in human osteosarcoma U2OS cells. Mechanistically, UFBP1 interacted with IkBα, regulated its stability, and thereby suppressed the NF-kB transcriptional activity [85]. UFL1 could alleviate the LPS-induced apoptosis in ovarian granulosa cells by regulating the NF-κB pathway, implying the role of UFL1 in the female reproductive system [86]. Importantly, p53 is traditionally regarded as a tumor suppressor. The alterations of the UFM1 modification system in various tumors are briefly summarized in Table 1.

## 5. Future Perspectives

Being a newfound UBL protein, UFM1 displays a conserved tertiary structure with ubiquitin. Its conjugation system is evolutionarily present among nearly all eukaryotic organisms, but not in yeast [2]. In contrast to the ubiquitin modification system consisting of varieties of E1, E2, and E3 enzymes, only one of each enzyme was identified in the UFM1 modification system [4]. Therefore, it remains elusive whether there exist additional E2s and E3s or other potential proteins. In addition, UFL1, the only E3 ligase identified in this system, does not possess any typical domains and seems to function merely as a scaffold protein, and the relationship between UFL1 and satellite components UFBP1 and CDK5RAP3 remains unclear.

Notably, multiple studies have demonstrated that the UFM1 modification system is implicated in numerous cellular functions and pathways, DNA damage response and erythroid development being among most well known in ER stress. Being involved in various cellular processes, it is unsurprising that the dysregulation of this modification leads to diseases such as cancer, diabetes, and heart failure. As we summarized, UFM1 modification is closely related to the pathogenesis and development of tumors, but it should be noted that this modification exerts a conflicting role depending on the cancer type. For instance, UFL1 exhibits a tumor suppressor role in hepatocellular carcinoma [43], while it functions as an oncogenic gene in lung cancer [81], suggesting that UFMylation might have conflicting consequences depending on the different cancer types or disease stages. In addition, the UFM1 cascade exerts an effect on a tumor mainly through modifying the substrate, and differences in substrates may partially explain the conflicting results. To date, the UFM1 modification system was demonstrated to be associated with a small group of tumors; whether it plays a role in other tumors, such as prostate cancer and esophageal carcinoma, is still far from being completely understood. Thus, the present findings on the mechanistic links between UFMylation and tumorigenesis represent only a starting point, and the potential and underlying mechanisms of UFMylation in the context of tumorigenesis need further studies and investigations; furthermore, whether UFM1 modification is related to other non-cancer diseases remains to be explored. In addition, although a few compounds were reported to inhibit the activity of the E1 enzyme UBA5, thus impeding the development of tumorigenesis [68,82,84], progress has stagnated due to the lack of suitable assay reagents necessary for their introduction. Therefore, the discovery of novel compounds or valid targets is an area of great interest that would paved the way for future cancer-related therapeutic interventions.

Lastly, as one of the PTMs, UFM1 modifies an array of substrates, with the currently known substrates being UFBP1 [40], ASC1 [41], p53 [62], RPL26 [75], RPN1 [87], MRE11 [59], and Histone H4 [61] (Table 2). Since this modification exerts an impact on cellular functions by influencing the substrate’s stability and biological functions, identifying new UFM1 substrates is of great importance. Innovative chemical tools as well as biochemical approaches should be employed. For instance, exploring antibodies recognizing the C-terminal Val-Gly motif of UFM1 coupled with innovative proteomics methodologies would improve the sensitivity and specificity for substrate detection. It is reported that the UFMylation of p53 stabilizes p53 by antagonizing its ubiquitination and proteasome degradation [62], implying a potential crosstalk between ubiquitination and UFMylation modification. The ways in which UFM1 affects modifications by other UBLs and the ways in which different modifications coordinate with each other merit detailed future investigations.

## 6. Conclusions

Generally, the UFM1 modification system and its biological functions have been well established and characterized. To date, emerging evidence has connected UFM1 to a wide range of human diseases. In this review, we summarize and detail the process and functions of UFM1 modification, mainly focusing on the specific roles and molecular mechanisms of the UFM1 modification system in the pathogenesis and development of various cancers, including breast cancer, gastric cancer, colon cancer, etc., in order to provide novel diagnostic biomarkers or therapeutic targets for these tumors. Nevertheless, whether the UFM1 modification system is involved in other tumors remains to be explored. Moreover, further in-depth investigations are urgently needed to unveil the other potential mechanisms. Finally, it is generally accepted that UFM1 exerts an impact through modifying certain substrates, thereby influencing the substrates’ stability, biological functions and interactions with target genes; thus, identifying new UFM1 substrates is an area of particular interest.

## Figures and Tables

**Table 1:** The role of UFM1 modification in various tumors.

Cancer	Gene Analyzed	Phenotype	Possible Mechanism	Ref.
Breast cancer	ASC1	UFMylation of ASC1 promoted the breast cancer cell growth and tumor formation	The polyufmylated ASC1 enhanced the association of p300, SRC1, and itself to the promoters of ERα target genes	[41]
	SLC7A11	Metformin suppressed tumor growth via reducing its stability	Metformin exerted anti-cancer effect in breast cancer by inhibiting the UFMylation of SLC7A11	[67]
	UBA5	Upregulated in breast cancer	Inhibitor-induced apoptosis, autophagy and ER stress in breast cancer cells	[68]
	LncUFC1	Upregulated in breast tissues and cell lines	Promoting the proliferation and migration of breast cancers via miR-34a/CXCL10 axis	[37]
Gastric cancer	UFM1	Upregulated in gastric tissues and cell lines	Suppressing gastric cancer development by attenuating the expression of PDK1	[69]
	UFBP1	High expression enhanced drug sensitivity in gastric cancer patients	Enhancing the sensitivity of gastric cancer cells to chemotherapy through the Nrf2/AKR1C axis	[71]
	CDK5RAP3	Low expression indicated a worse outcome of gastric cancer patients	Suppressing the development of gastric cancer via inhibiting Akt/GSK-3β and Wnt/β-catenin signaling	[72,73]
	LncUFC1	Downregulated in gastric tissues	Promoting gastric cancer development by regulating miR-498/Lin28b pathway	[38]
Colon cancer	UFSP2	Decreased in colon cancer patients	Suppressing the growth rates of colon cancer cells and xenograft tumors	[74]
	LincUFC1	Overexpressed in colorectal tissues	Promoting the colorectal cancer growth by regulating the β-catenin and p38 signaling	[35]
Hepatocellular carcinoma	UFM1	Decreased in hepatocellular carcinoma tissues	Direct mechanism unknown	[77]
	UFL1	Detected in hepatocellular carcinoma cell line	Preventing cell invasion, inhibiting NF-kB signaling	[43]
	CDK5RAP3	Controversial	Controversial	[78,79,80]
Lung cancer	UFL1	Upregulated in lung adenocarcinoma tissues	Inhibiting the ubiquitin-mediated proteasome degradation of p120 catenin	[81]
	UBA5	Design an UBA5 inhibitor	Inhibitor reduced the proliferation of lung cancer cells	[82]
	CDK5RAP3	Elevated in lung adenocarcinoma tissues	Unknown	[83]
Pancreatic cancer	UBA5	Chemoproteomic screening identified	Knockdown impaired pancreatic cancer pathogenicity	[84]
Osteosarcoma	UFBP1	Depletion inhibited cell proliferation and invasion	Suppressing the NF-kB transcriptional activity	[85]
Ovarian granulosa cells	UFL1	Alleviating the LPS-induced apoptosis in ovarian granulosa cells	Regulating the NF-κB pathway	[86]

**Table 2:** Summary of the identified UFMylated substrates.

Substrate	Modification Sites	Function after UFMylation Modification	Ref.
UFBP1	Lys267	Maintaining ER homeostasis	[40]
ASC1	Lys324, Lys325, Lys334 and Lys367	Transactivation of ERα and promoting breast cancer development	[41]
p53	Lys351, Lys357, Lys370 and Lys373	Maintaining p53 stability and suppressing tumor progression	[62]
RPL26	Lys132 and Lys134	Protein biogenesis at the ER.	[75]
RPN1	Unknown	ER phagy	[87]
MRE11	Lys282	Promoting ATM activation, DSB repair and genome stability	[59]
Histone H4	Lys31	Promoting ATM activation and maintaining genomic integrity	[61]

ER: endoplasmic reticulum; ATM: ataxia–telangiectasia mutated; DSB: double strand breaks.

## Abbreviations

AKR1C	Aldo-keto reductase 1C
ASC1	Activating signal co-integrator 1
ATF6	Activating transcription factor 6
ATM	Ataxia–telangiectasia mutated
CDK5	Cyclin-dependent kinase 5
DDR	DNA damage response
DSBs	Double-strand breaks
DUBs	Deubiquitinating enzymes
ER	Endoplasmic reticulum
ERα	Estrogen receptor-α
ERAD	ER-associated degradation
HCC	Hepatocellular carcinoma
HR	Homologous recombination
HSC	Hematopoietic stem cell
IRE1α	Inositol-requiring enzyme 1α
lincRNA	Long intergenic RNA
LncRNA	Long non-coding RNA
NHEJ	Non-homologous end-joining
NLS	Nuclear localization signal
PERK	Protein kinase-like ER kinase
PTMs	Post-translational modifications
RING	Really interesting new gene
SCNAs	Somatic copy number alterations
UBA5	Ubiquitin-like modifier-activating enzyme 5
UBLs	Ubiquitin-like molecules
UFC1	UFM1-conjugating enzyme 1
UFL1	UFM1-specific ligase 1
UFM1	Ubiquitin-fold modifier 1
ULPs	Ubiquitin-like protein-specific proteases
UPR	Unfolded protein response
